# Optimizing the Parameters of Vagus Nerve Stimulation by Uniform Design in Rats with Acute Myocardial Infarction

**DOI:** 10.1371/journal.pone.0042799

**Published:** 2012-11-26

**Authors:** Shan-Shan Kong, Jin-Jun Liu, Tyzh-Chang Hwang, Xiao-Jiang Yu, Mei Zhao, Ming Zhao, Bing-Xiang Yuan, Yi Lu, Yu-Ming Kang, Bing Wang, Wei-Jin Zang

**Affiliations:** 1 Department of Pharmacology, Xi'an Jiaotong University, College of Medicine, Xi'an, People's Republic of China; 2 Department of Medical Pharmacology and Physiology, and Dalton Cardiovascular Research Center, University of Missouri-Columbia, Columbia, Missouri, United States of America; 3 Department of Physiology and Pathophysiology, Xi'an Jiaotong University, College of Medicine, Xi'an, People's Republic of China; 4 Department of Pathology, Xi'an Jiaotong University, College of Medicine, Xi'an, People's Republic of China; University of California, Los Angeles, United States of America

## Abstract

Vagus nerve stimulation (VNS) has been shown to improve left ventricular function and survival in rats with acute myocardial infarction (AMI), and this maneuver has also been adopted clinically for the treatment of patients with chronic heart failure (CHF). Recent in vitro and in vivo studies have suggested that VNS can modulate the level of pro-inflammatory factors. Despite the beneficial effects of VNS, the stimulation parameters for obtaining favorable outcomes appear highly variable. To optimize VNS parameters, we set up different stimulation protocols with different pulse width (1–2 ms), frequency (1–6 Hz), voltage (1–6 V) and duration (40–240 min) of VNS by uniform design (UD). Rats were divided into seven groups with (Group1–Group6) or without VNS (MI group). Our results demonstrate that (1) the parameter sets in Group1, Group2 and Group3 yield the best post-MI protection by VNS, while the protective role were not observed in Group4, Group5 and Group6; (2) baroreflex sensitivity and the α7 nicotinic acetylcholine receptor level were also increased in Group1, Group2 and Group3. (3) the parameter set in Group1 (G1:1 ms, 2 Hz, 3 V, 240 min) is judged the most optimal parameter in this study as rats in this group not only showed a reduced myocardial injury with better-preserved cardiac function compared with other groups, more important, but also exhibited minimal heart rate (HR) reduction. (4) the duration of VNS plays an important role in determining the protection effect of VNS. In conclusion, VNS displays a beneficial role in Group1, Group2 and Group3. Of note, the parameter set in Group1 provides the most optimal cardioprotective effect. These results may provide insight into development of novel treatment for ischemic heart diseases.

## Introduction

The autonomic nervous system (ANS), divided into the sympathetic and parasympathetic (vagal) subsystems, plays an important role in the regulation of the mammalian heart. High cardiac sympathetic tone and reduced vagal activity are not only the characteristic autonomic phenotype associated with myocardial infarction (MI) and chronic heart failure (CHF) but also an independent predictor of mortality [1], [2]. Thus the cardiac vagal activity maybe is a target for therapeutic interventions in cardiovascular diseases.

The vagus nerve originates from the medulla and innervates organs of the neck, thorax as well as abdomen. However, the left and right efferent vagal nerves play different roles in controlling mammalian heart. Cardiac efferents in the left vagus nerve are involved in modulating the conductivity of the atrioventricular node and regulating cardiac contractility, while cardiac efferents in the right vagus nerve regulate the heart rate (HR) through the sinoatrial node [3], [4]. Recent experimental evidence from several laboratories including our own has shown that electrical stimulation of the right efferent vagal nerve improves the cardiac function in rats with acute myocardial infarction (AMI) [5] and modulates left ventricle remodeling and markedly improve survival after MI in rats [6]. In patients with heart failure, this maneuver has been shown to also improve cardiac performance suggesting an ameliorative effect of direct neural interventions against ischemia heart diseases [7]. Therefore, augmenting vagal activity by vagus nerve stimulation (VNS) has potential beneficial effect on patients with ischemic heart disease.

In addition to the aforementioned roles of the autonomic nerve system in cardiac pathophysiology, recent studies also suggest an involvement of pro-inflammation factors in the progression of ischemic heart disease, especially in AMI and CHF. For example, an elevated level of tumor necrosis factor-α (TNF-α) was directly associated with the deterioration of cardiac functional class and left ventricular ejection fraction [8], [9]. The interesting connection between the autonomic system and the immune system was reported by Borovikova *et al.* which was named the cholinergic anti-inflammatory way [10]. They found that VNS can modulate the release of TNF-α and its expression, mediated chiefly via the α7 nicotinic acetylcholine (ACh) receptor [11]. This observation was also confirmed repeatedly by others [12], [13], [14].

Despite these well-documented beneficial effects of VNS on cardiac diseases, the stimulation parameters for obtaining optimal outcomes appear highly variable in different model systems [5], [6], [12], [15], [16], [17] (Table 1). In the present study, in an attempt to optimize VNS parameters we employed the method of uniform designs (UD) proposed by Fang [18]. The major advantage of the UD method is that the number of experiments is considerably less than that required by other known experimental-design techniques when the number of levels of factors is large. In this study, an optimal set of VNS parameters that maximally preserve cardiac function was attained. The clinical implication of our results will be discussed.

**Table 1:** Parameters of vagus nerve stimulation on rats with ischemia heart disease.

Model	Pulses width (ms)	Frequency (Hz)	Intense (V)	Duration (min)	References
Myocardial infarction	1	5	2–6	240	[5]
Heart failure	0.2	20	unknown	240/day,6w	[6]
Ischemia Reperfusion	2	10	unknown	10	[12]
Ischemia-induced arrhythmia	0.1	10	2–6	30	[15]
Ischemia Reperfusion	2	1–9	5	7or10	[16]
Myocardial ischemia	0.1	10	unknown	180	[17]

## Methods

### Animals

For this experiment, male adult Sprague-Dawley rats weighing 200–250 g (supplied by the Experimental Animal Center of Xi'an Jiaotong University, China) were used in accordance with recommended guidelines on the care and use of laboratory animals issued by the Chinese Council on Animal Research. The study was approved by the ethics committee at Xi'an Jiaotong University.

### Hemodynamic measurements

Anesthesia was induced by intravenous injection of pentobarbital sodium (35 mg/kg). Rats were tracheotomized, intubated, and mechanically ventilated. (Arterial pH, PO_2_ and PCO_2_ were maintained within the physiological ranges by supplying oxygen and changing the respiratory rate). A heparin-filled polyethylene catheter (PE-50) was placed in the left ventricle via the right carotid artery to measure left ventricular end-diastolic pressure (LVEDP) and maximum rate of increase/decrease rate of left ventricular pressure (± dP/dt max). Another heparin-filled polyethylene catheter (PE-50) was inserted into the right femoral artery for the recording of the systolic blood pressure (SBP) and diastolic blood pressure (DBP). An instantaneous HR was measured from the lead II electrocardiogram (ECG). Signals were recorded with Chart v (Powerlab, AD Instruments, Australia) throughout the experiment until 5 min before the end.

### Baroreflex sensitivity measurements

Thirty minutes were allowed for stabilization after the initial preparation and surgical procedures were completed. Baroreflexes were elicited by bolus injection of phenylephrine (5 µg/kg, i.v.) via the right femoral vein [19]. Baroreflex sensitivity was calculated as the slope of the linear portion of the relationship between the R-R interval (in ms) and the mean arterial pressure (in mmHg). Only animals with a correlation coefficient *R*>0.8 and a *P* value<0.05 were included into the study.

### Acute myocardial infarction model and vagus nerve stimulation

AMI was produced by 4 h of the left anterior descending (LAD) coronary artery ligation as previously described [20]. Briefly, left thoracomy and pericardiectomy were performed, and the hearts were gently exteriorised. LAD was ligated ∼3 mm below the left atrium with a 5-0 silk suture. The chest wall was then closed. Acute myocardial ischemia was deemed successful on the basis of regional cyanosis of the myocardial surface distal to the suture, accompanied by elevation of the ST segment on ECG [21].

**Figure 1:**
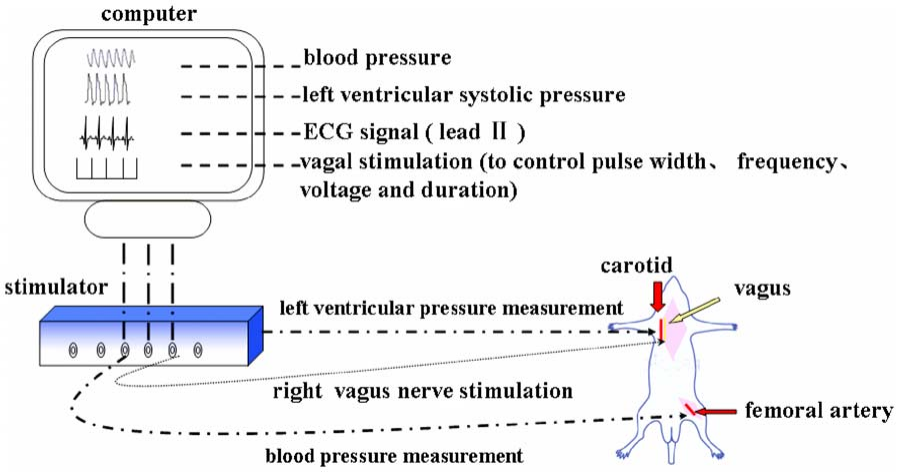
The schematic representation of the experimental set up. During the experiment, the distal end of right vagus nerve was stimulated. A polyethylene catheter (PE) catheter was inserted into the left femoral artery to monitor the blood pressure. Another PE catheter was inserted into the right carotid artery to measure the changes of left ventricular pressure. The intense of vagal stimulation was controlled by the computer which was connected with the simulator. The signal of vagal stimulation was output by the stimulator and was displayed in the computer.

Bilateral cervical vagus nerves were identified and transected at the neck region. Following the previous experience [5], [6], [7], [22], [23], only the cardiac end of the right vagus nerve was stimulated to exclude the effects of the vagal afferent. Under general anesthesia (sodium pentobarbital, 35 mg/kg) and mechanical ventilation, the distal segments were fixed on a with a Teflon-coated silver wire electrode (0.1 mm diameter), connected to the output terminals of the isolated constant voltage stimulator (ML866, Powerlab, AD Instruments, Australia) which allowed stimulation over a range of frequencies (0.1–100 Hz) and strengths (1–10 V) as well as pulse width (0.001–10 s.) (Figure 1). VNS were performed immediately after the LAD artery ligation. To prevent drying and to provide insulation, the stimulation electrodes and the vagus nerve were immersed in a mixture of white petrolatum (Vaseline) and paraffin [24], [25], [26].

### Uniform design and Experimental protocol

UD tables can be expressed as Un(t^s^), where U stands for the UD, n for the number of experimental trials, *t* and *s* is for the number of levels and the maximum number of factors, respectively. Previous study has shown that several factors have significant effects on the role of VNS including pulse width, frequency, pulse voltage and the duration of VNS [23]. Thus the experiment for optimization of VNS was arranged as four factors and each was at six levels. In the present study, the UD table U_6_ (6^4^) was applied to arrange the experiments.

**Figure 2:**
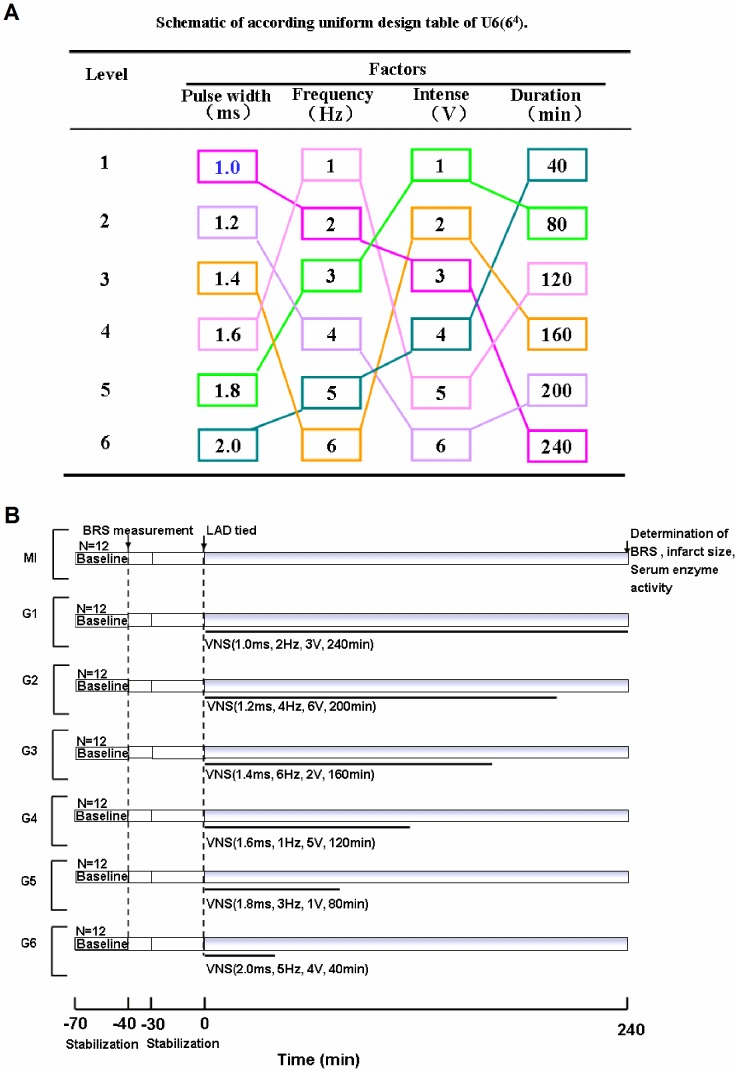
Uniform design protocol and schematic representation of the protocols of study. (A) Factors and levels in the uniform design experiments. (B) Experiment of protocol. After initial surgical preparation, all rats were allowed 30 minutes (min) stabilization. Rats in each group were then treated a bolus injection of phenylephrine (5 µg/kg, i.v.) via the right femoral vein to measure baroreflex sensitivity (BRS). After this, all rats wer*e* allowed to stabilize over a 30-minute period before left anterior descending (LAD) artery ligation. Acute myocardial infarction (AMI) was induced by 240 min of LAD artery ligation. In MI group, the rats were just received LAD without VNS. In the G1–G6 group, vagus nerve stimulation (VNS) was performed immediately after the LAD artery ligation. Different vagal stimulation parameters following LAD artery ligation was also demonstrated in experiment protocol.

All rats were randomly allocated to one of seven groups which were undertaken LAD occlusion for 240 min. In MI group, rats were only undertaken the LAD occlusion for 240 min without vagal stimulation. In the other six groups, the rats were treated with different VNS parameters. As shown in Figure 2, the rats were divided into these groups (G1–G6) based on differences in pulse width, frequency, voltage, and duration of stimulation. Each trial was performed in triplicate.

### Exclusion criteria

Rats were excluded from the study when the following criteria were observed: 1) the rats died during the surgical procedures; 2) intractable severe arrhythmia occurred and/or responded poorly to VNS; 3) a sustained fall in mean arterial pressure (MAP) less than 60 mmHg [5].

### Infarction area assessment

At the end of experiment, 2 ml of 5% Evans Blue was injected slowly to the vena cava and used to determine the left ventricular tissue that was not subjected to regional ischemia. The myocardial ischemic area at risk (IAR) was identified as the region without any blue stain. Then the heart was immediately sliced into seven 2-mm thick sections and these were incubated with 5% 2,3,5-triphenyltetrazolium chloride (TTC) at room temperature for 30 min. The sections then transferred into 10% formalin and kept at 4°C for 48 h. The infarction size was defined as TTC-unstained area and expressed as the percentage of the IAR [5].

### Biochemical assay and TNF-α measurement

At the end of experiment, blood samples from some rats (2 mL whole blood) were collected to measure the myocardial leakage including lactate dehydrogenase (LDH), creatine kinase (CK) and cardiac troponin T (cTnT) as well as TNF-α level. Serum was obtained by centrifugation at 6000 g for 6 min [27]. Serum LDH and CK activity were analyzed according to the manufacture's instructions (NanJing Jiancheng Biological Technology, NanJing, China) and the activity of LDH and CK were expressed as U/L. Serum level of cTnT was measured with electrochemiluminescence technology (third-generation cardiac TnT, Elecsys 2010, Roche, Mannheim, Germany). Serum level of TNF-α was measured by a rat enzyme-linked immunosorbent assay (ELISA) kit (R&D Systems, Minneapolis, MN, USA), according to the manufacture's instructions. The concentration of TNF-α was calculated from a standard curve constructed using the standards supplied with the kits. All samples and standards were measured in triplicate.

### Western blot

As described previously [28], protein samples (80 µg) were separated on 12% sodium dodecyl sulphate olyacrylamide gels and transferred onto a polyvinylidene difluoride (PVDF) membrane (Bio-Rad, Hercules, CA, USA). Membranes were blocked with 5% non-fat dry milk in Tris-buffered saline containing 0.1% Tween 20 (TBST) and incubated with primary antibodies as following: goat anti-TNF-α polyclonal antibodies (diluted 1∶ 200; Santa Cruz Biotechnology, Santa Cruz, CA, USA), rat anti-α7 (diluted 1∶ 200; Santa Cruz), and mouse anti-GAPDH (1∶ 5000; Advanced Immunochemical, Long beach, CA, USA). GAPDH expression was used as an internal control. After washing three times with TBST, the membranes were incubated with horseradish peroxidase conjugated rabbit anti-goat IgG (diluted 1∶ 1000; Santa Cruz), mouse anti-rat IgG (diluted 1∶ 1000; Santa Cruz) and goat anti-mouse IgG (diluted 1∶ 1000; Santa Cruz). Chemiluminescence was detected using an ECL-Plus kit (PerkinElmer Life Science, Waltham, MA, USA) and the light signals were detected by X-ray film.

### Statistical analysis

Data are expressed as mean ± SEM. *P*<0.05 was considered significant. Results were compared using one-way ANOVA followed by Tukey's multiple comparison test. Dunnett's test was used to compare the difference for the TNF-α ELISA measurement.

## Results

### Effect of VNS on the HR in rats with AMI

**Figure 3:**
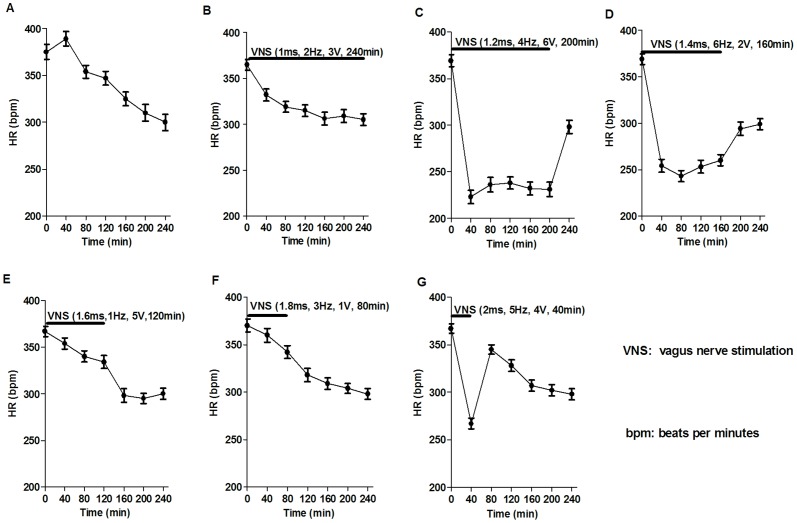
Time courses of heart rates (HR) change during acute myocardial infarction in rats with or without vagal simulation. The baseline values obtained just before LAD ligation are shown at time 0. (A) HR change in rats with AMI. Myocardial ischemia alone caused an increase initially then a progressively decrease in HR. (B–G) HR change in rats during AMI with vagal stimulations. HR was reduced from 1% to 45% compared to the baseline level. After the cessation of vagal stimulations, HR tends to increase back to the baseline level (n = 12 for each group).

Figure 3 showed typical HR changes in rat during AMI with or without vagal stimulations. The degree of HR reduction varied considerably following different VNS interventions. In the MI group wherein rats were only subjected to LAD, HR increased slightly at the early phase of the experiment, but decreased later as the experiment progressed. This result is in line with our previous report. The highest (45%) and the lowest (1%) HR reductions were observed in G2 (1.2 ms, 4 Hz, 6 V, 200 min) and G5 (1.8 ms, 3 Hz, 1 V, 80 min) respectively. In G3 (1.6 ms, 6 Hz, 2 V, 160 min) and G6 (2.0 ms, 5 Hz, 4 V, 40 min), a similar HR reduction (20∼25%) was seen despite differences in the duration of VNS. In G1 (1 ms, 2 Hz, 3 V, 240 min), VNS caused ∼10% HR reduction and in G4 VNS led to ∼5% HR reduction. After the cessation of VNS, HR tends to increase toward the baseline level, an indication of the effectiveness of VNS.

### Effects of VNS on serum enzymes and myocardial infarction size

**Figure 4:**
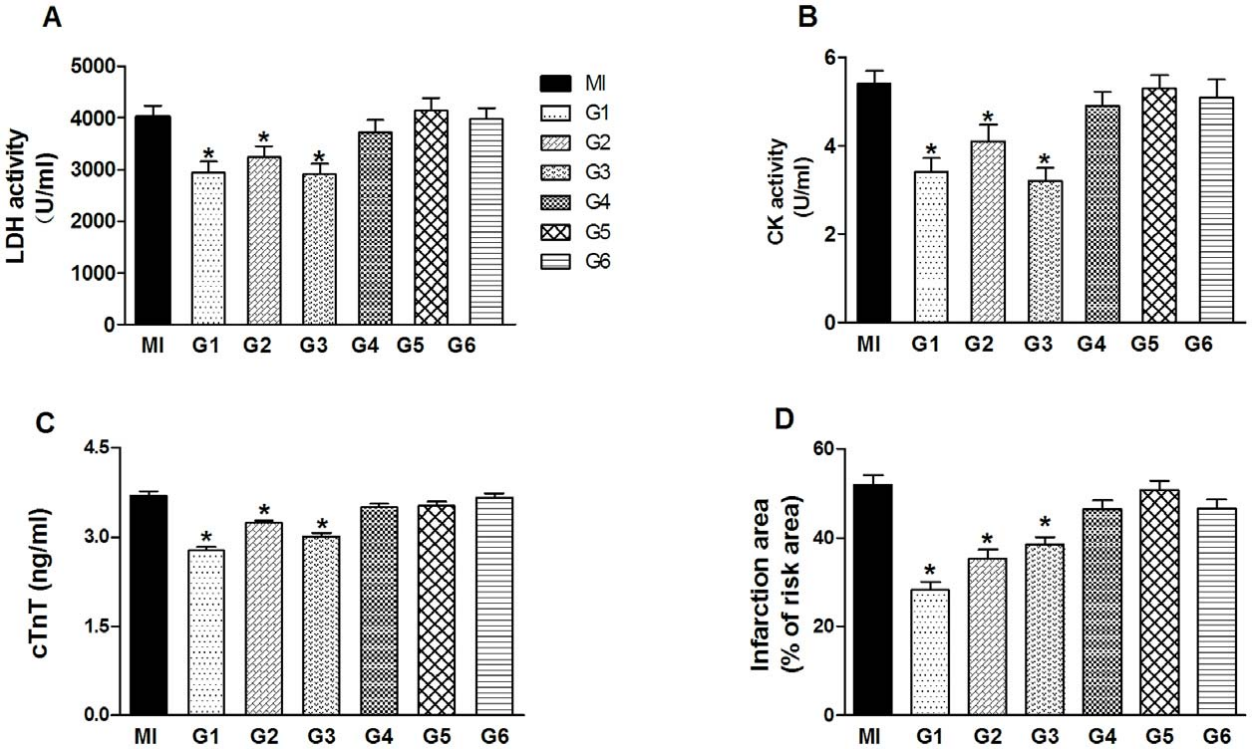
Effects of vagal stimulation on serum enzyme activity and infarction size following acute myocardial infarction in MI and G1–G6 group. (A) Lactate dehydrogenase (LDH), (B) creatine kinase (CK), (C) cTnT activity in the serum (n = 8 for each group). (D) Changes of myocardial infarction size (percentage risk area) by VNS. (n = 8 for each group). Data are expressed as mean ± SEM Data are the mean ± SEM. ^*^ *P*<0.05 compared with MI control group.

Figure 4A–4C shows serum levels of three cardiac enzymes (LDH, CK and cTnT) in all groups. When compared to controls, only in G1, G2 and G3, the changes in serum LDH, CK and cTnT reached statistical significance. Interestingly, these three groups were subjected to longer durations of VNS and thus exhibited a prolonged reduction of HR. The importance of the duration of VNS is best demonstrated when compared results between G1, G2 and G3. While there was a compatible HR reduction between G3 and G6, the duration of VNS for G6 is much shorter (40 min). On the other hand, although the magnitude of HR reduction was moderate in G1, VNS did significantly lower the serum levels of LDH and CK as well as cTnT.

We next assessed the protective role of VNS by examining directly the effects of VNS on myocardial infarction size. Figure 4D shows quantitative comparison of the infarction area among these groups. Interestingly, the protection effect of VNS on the infarction size almost parallels those changes of serum enzymes shown in Figure 4A–4C as expected since these proteins are released from infarcted myocardium.

### Effects of VNS on hemodynamics

**Figure 5:**
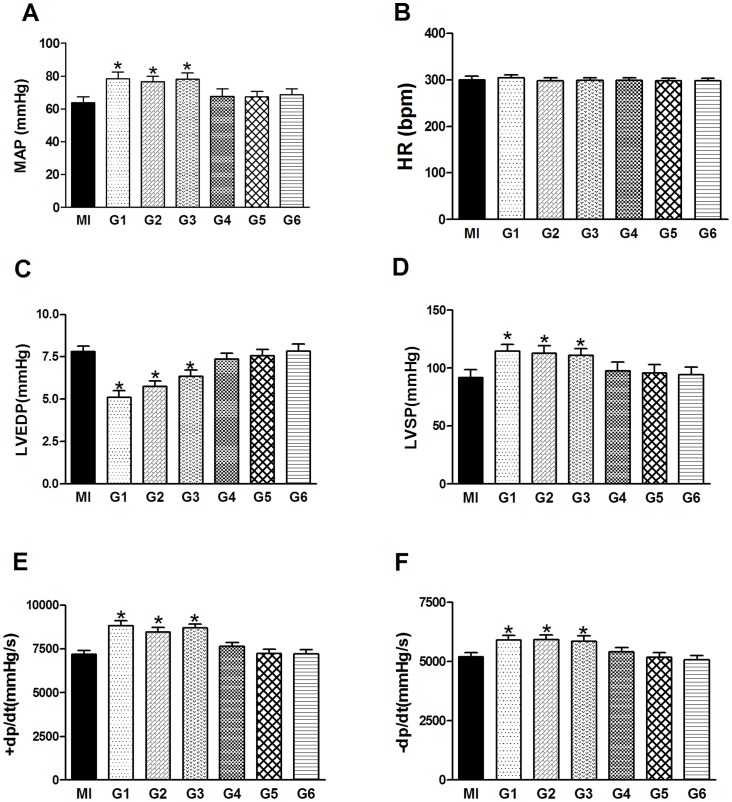
Effects of vagus nerve stimulation (VNS) on cardiac function in MI and G1–G6 group. MAP, mean arterial pressure; HR, heart rate; LVSP, left ventricular systolic pressure; LVEDP, left ventricular end-diastolic pressure; ±dP/dt, maximum slope of systolic pressure increment and diastolic pressure decrement. Data are the mean ± SEM (n = 8 for each group). ^*^ *P*<0.05 compared with MI control group.

The effects of VNS on hemodynamics were measured before 5 min of the end of experiments (Figure 5). Compared with the MI group, rats in G1, G2 and G3 groups exhibited significant improvement in cardiac function after AMI, which manifested a higher MAP, lower LVEDP and an increased maximum dP/dt of left ventricular (LV) pressure. However, there was no improvement in cardiac function in G4, G5 and G6 compared to MI group.

### Effects of VNS on the expression of TNF-α

**Figure 6:**
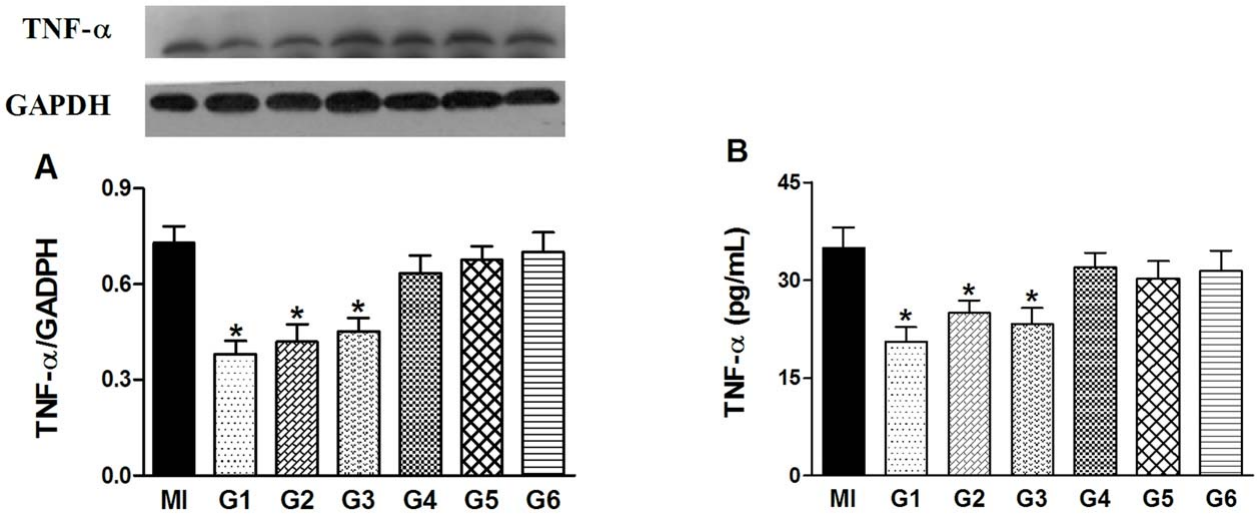
Effects of vagus nerve stimulation on myocardial and plasma level of tumor necrosis factor-α (TNF-α) in MI and G1–G6 group. (A) Representative blots and densitometric analyses of TNF-α protein expression, normalized against GADPH. (B) Arterial blood levels of TNF-α, as determined by ELISA. Data are the mean ± SEM (n = 8). ^*^ *P*<0.05 compared with MI group.

We measured levels of TNF-α to see if these different VNS protocols yield different levels of inhibition and if the effect of VNS on the expression of TNF-α is in agreement with the effects shown in Figure 4. Figure 6 shows the representative Western blots for TNF-α in the infarcted myocardium (Figure 6A) as well as the serum levels of TNF-α (Figure 6B). Although compared with the control rats, all treatment groups showed some reduction of TNF-α, only in G1, G2 and G3 the reduction of the TNF-α level reached statistical significance in both Western blots and ELISA assay.

### Effects of VNS on BRS

**Figure 7:**
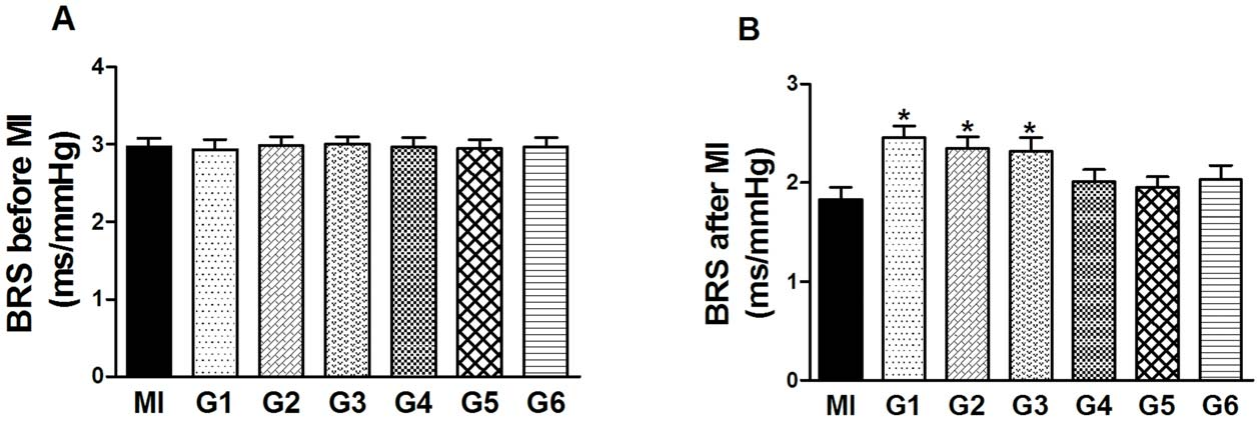
Impacts of vagal stimulation on baroreflex sensitivity (BRS) change. BRS was quantitated as slope of a line generated from the relationship of R-R interval during increases in arterial pressure. (A) The BRS baseline level in each group at the stabilization period. (B) The BRS levels in each group at the end of experiment. The levels of BRS were obviously higher in G1, G2 and G3 compared with the MI group. Data are the mean ± SEM (n = 8). ^*^ *P*<0.05 compared with MI group.

The baroreceptor reflex response was quantified as described in Materials and Methods. It was obtained from rats during the stabilization and the AMI period with or without VNS, respectively. Rats in each group had almost the similar levels of BRS before different interventions (Figure 7A). At the end of experiments, BRS was measured with the same method again. Figure 7B demonstrates the increased BRS level in G1, G2 and G3 when compared to the control MI group.

### Effects of VNS on α7-nicotinic ACh receptor expression

**Figure 8:**
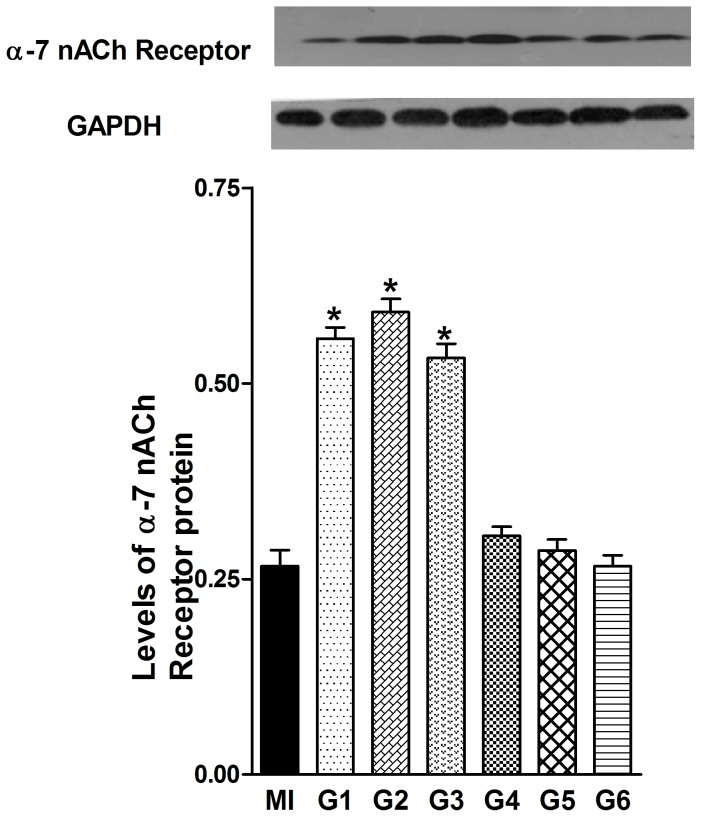
Representative blot and densitometric analyses of α7-nicotinic receptor protein from the myocardial. The levels of α7-nicotinic receptor were increased significantly in G1, G2 and G3 compared with the MI group. Data are the mean ± SEM (n = 8). ^*^ *P*<0.05 compared with MI group.

Since it has been reported that the nicotinic ACh receptor α7 subunit is required for the anti-inflammatory role of vagus nerve, we next determined the expression of α7-nicotinic ACh receptor in the infarcted myocardium with Western blot analysis (Figure 8). Similar to the observation made for TNF-α expression, the levels of α7-nicotinic ACh receptor in G1, G2 and G3 were altered to a significant amount when compared to the MI group. In contrast, compared with MI group, G4, G5 and G6 did not show significant differences in the expression of α7-nicotinic ACh receptor.

### Parameter Analysis for the vagal stimulation by DAS3.0 software

To analyze which factor (pulse width, frequency, voltage and stimulation duration) is important in affecting the protective role of VNS, we used DAS 3.0 software to assess the vagal stimulation parameter. The results demonstrated that (1) the duration of stimulation plays an important role in deciding the cardioprotective role of VNS. (2) the optimal mixture ratio among each factor is 1∶6∶6∶240 (pulse width∶ frequency∶ voltage∶ duration). (3) the frequency and duration present a synergistic effect; however, the pulse width and duration present an antagonist effect.

## Discussion

Despite the beneficial effects of VNS, the stimulation parameters for obtaining favorable outcomes appear highly variable. To optimize VNS parameters, in the present study, we designed, for the first time, 6 different parameter sets for VNS in rats with AMI by UD method and examined their effects on conventional MI indexes such as serum LDH, CK and cTnT, and infarction size as well as newly developed index, serum level of TNF-α. Our results displayed that (1) the duration of VNS plays a more important role in determining the protection effect of VNS; (2) parameter set in G1 (1 ms, 2 Hz, 3 V, 240 min) provides an optimal protection as judged by a better-preserved cardiac function and moderate HR reduction (10%).

It has been reported that VNS has therapeutic effect on human CHF and rats with AMI. These studies have shown that several factors determine the effectiveness of VNS including pulse width, frequency, pulse voltage and the duration of VNS. However, it is very difficult, if not impossible, to analyze and assess the effect of these factors on cardioprotective role of VNS by a full factorial design (FD) because there will be 7^4^ = 2401 experiments for a seven-level FD design. Even if we choose orthogonal design (OD), it still needs 7^2^ = 49 experiments for a seven-level FD design. In contrast, 7 experiments will be sufficient for a seven-level UD design. Thus, UD has the least number of experiments, and thereby can significantly reduce the expense and time of experimentation.

In this study, our results demonstrated the rats in G1, G2 and G3 were effectively protected by VNS but not in G4, G5 and G6. Although the experimental protocol for G2 caused the highest HR reduction (45%), it did not elicit the best beneficial effect on the cardiac function, serum enzyme or myocardial infarction size, a prolonged, more drastic HR reduction may compromise cardiac output and therefore impose an opposite effect. A reduction of HR may help minimize the infarction size as demonstrated in G3, where a 20%∼25% reduction in HR was observed. Of note, an effective protection can be attained in G3, but the magnitude of HR reduction is perhaps clinically infeasible. In contrast, rats in G1, exhibiting only 10% HR reduction, manifested the best protection of VNS. This includes an improvement of cardiac function in contractility (+dP/dt max) and diastolic pressure (LVEDP), a lowering of serum enzymes and myocardial infarction size. Thus, the protective effect of VNS on MI-induced cardiac injury does not require a large scale of HR reduction, a finding consistent with previous reports on animal models of mice [29] and rats [6], as well as on patients with heart failure [7].

Despite different parameter sets of VNS, one common feature among protocols used in G1, G2, and G3 is the longer duration of VNS compared with those used for rats in G4, G5, and G6. The importance of a sustained duration of HR reduction is further affirmed by the observation that rats in G6 did not exhibit significant cardioprotection even though a 20∼25% HR reduction was seen. It is in line with the results analyzed by DAS3.0 software that stimulation duration is an important factor in determining the protective role of VNS. It should be noted that although the current study shows a correlation between HR reduction and cardioprotection, this relationship may not be causal as demonstrated in Katare *et al* [30], wherein cardioprotective effect of VNS was seen with minimal drop of HR. Thus, factor(s) other than a decrease of HR may also be involved in VNS-induced cardioprotection. One possible contributing factor entails molecules involved in inflammatory reactions such as TNF-α. It has been reported that levels of TNF-α in the heart are increased within 15 min of ischemia, whereas TNF-α mRNA levels increase even earlier [31] and persist in cardiomyocytes with time [32]. Irwin *et al.* reported that an increase of the TNF-α level in the infarcted myocardium can increase the TNF-α level in the peri-infarct myocardium, leading to an amplified cytokine effect [32]. Furthermore, TNF-α contributes significantly to cardiac dysfunction and inflammatory responses to MI play a significant role in determining infarct size [33], [34]. Figure 5 depicted that TNF-α was increased in MI group whereas TNF-α was significantly lowered by VNS in G1, G2 and G3. These findings are in accord with those published previously [5], [35].

In a more recent study by Wang *et al.*, VNS inhibits TNF-α release and protects against systemic inflammation through a mechanism dependent on the α7 nicotinic acetylcholine receptor [11]. In the current study, we detected a significant increase in the expression of α7 nicotinic acetylcholine receptor in G1, G2 and G3 rats compared to the MI rats. Furthermore, the BRS measurements confirmed that the vagal tone was indeed increased in the G1, G2 and G3 rats. Based on these findings, we propose that the increased vagal tone in G1, G2 and G3 may play a vital role in benefiting the rats with AMI.

In summary, our results suggested that (1) stimulation duration of VNS plays an important role in determining the cardioprotective role of VNS; (2) the parameter set in G1 is the most optimal condition in this study. Our results might provide information to guide selection of stimulation parameters for therapeutic applications of VNS in patients with myocardial ischemia. As the current report as well as previous work [5] suggests a role of TNF-α in the pathogenesis of AMI, future studies on the mechanisms underlying the anti-inflammation action of VNS could pave the way for the development of new strategies in the treatment of AMI.
